# Post-mortem Plasma Cell-Free DNA Sequencing: Proof-of-Concept Study for the “Liquid Autopsy”

**DOI:** 10.1038/s41598-020-59193-y

**Published:** 2020-02-07

**Authors:** Erina Takai, Daichi Maeda, Zhuo Li, Yukitsugu Kudo-Asabe, Yasushi Totoki, Hiromi Nakamura, Akiko Nakamura, Rumi Nakamura, Misato Kirikawa, Yukinobu Ito, Makoto Yoshida, Takamitsu Inoue, Tomonori Habuchi, Shohei Ikoma, Hiroto Katoh, Mamoru Kato, Tatsuhiro Shibata, Shumpei Ishikawa, Shinichi Yachida, Akiteru Goto

**Affiliations:** 1 Department of Cancer Genome Informatics, Graduate School of Medicine, Osaka University, Osaka, Japan; 2 Department of Clinical Genomics, Graduate School of Medicine, Osaka University, Osaka, Japan; 3 Department of Cellular and Organ Pathology, Graduate School of Medicine, Akita University, Akita, Japan; 4 Department of Laboratory Medicine, The First Affiliated Hospital of Xi’an Medical University, Xi’an, Shaanxi P. R. China; 5 Division of Cancer Genomics, National Cancer Center Research Institute, Tokyo, Japan; 6 Faculty of Medicine, Akita University, Akita, Japan; 7 Department of Urology, Graduate School of Medicine, Akita University, Akita, Japan; 8 Department of Pathology and Laboratory Medicine, David Geffen School of Medicine, University of California, Los Angeles, CA USA; 9 Department of Genomic Pathology, Medical Research Institute, Tokyo Medical and Dental University, Tokyo, Japan; 10 Department of Preventive Medicine, Graduate School of Medicine, The University of Tokyo, Tokyo, Japan; 11 Department of Bioinformatics, National Cancer Center Research Institute, Tokyo, Japan; 12 Laboratory of Molecular Medicine, Human Genome Center, The Institute of Medical Science, The University of Tokyo, Tokyo, Japan

**Keywords:** Molecular medicine, Clinical genetics, Cancer, Cancer genomics, Tumour heterogeneity, Urological cancer

## Abstract

Recent genomic studies on cancer tissues obtained during rapid autopsy have provided insights into the clonal evolution and heterogeneity of cancer. However, post-mortem blood has not been subjected to genetic analyses in relation to cancer. We first confirmed that substantial quantities of cell-free DNA were present in the post-mortem plasma of 12 autopsy cases. Then, we focused on a pilot case of prostate cancer with multiple metastases for genetic analyses. Whole-exome sequencing of post-mortem plasma-derived cell-free DNA and eight frozen metastatic cancer tissues collected during rapid autopsy was performed, and compared their mutational statuses. The post-mortem plasma cell-free DNA was successfully sequenced and 344 mutations were identified. Of these, 160 were detected in at least one of the metastases. Further, 99% of the mutations shared by all metastases were present in the plasma. Sanger sequencing of 30 additional formalin-fixed metastases enabled us to map the clones harboring mutations initially detected only in the plasma. In conclusion, post-mortem blood, which is usually disposed of during conventional autopsies, can provide valuable data if sequenced in detail, especially regarding cancer heterogeneity. Furthermore, post-mortem plasma cell-free DNA sequencing (liquid autopsy) can be a novel platform for cancer research and a tool for genomic pathology.

## Introduction

Evidence of the clonal evolution of cancer is rapidly accumulating, and tumor heterogeneity is now recognised as a critical issue in the era of personalised cancer medicine^1,2^. To precisely evaluate the clonal evolution and heterogeneity of cancer, sequence analysis of multiple cancer lesions is essential. Considering the difficulties associated with sampling multiple cancer tissues from living patients, particularly from those who have undergone chemo- or immunotherapy, autopsies are highly valuable. In fact, several rapid autopsy studies have led to important findings in the fields of prostate cancer, pancreatic cancer, renal cell carcinoma, and breast cancer^3–6^. However, the genotypes of cell-free DNA (cfDNA) in post-mortem blood samples have never been analyzed in relation to cancer.

Circulating cfDNA from cancer patients contains tumor DNA (circulating tumor DNA); therefore, sequencing analyses of cfDNA from cancer patients provide information about genomic changes in tumors within a patient without surgical resection or tissue biopsy^7,8^. The identification of cancer-associated gene mutations in plasma now comprises the main part of the “liquid biopsy,” a novel method for cancer detection and monitoring^7,8^. Analyses of cfDNA are expected to provide an overview of the somatic changes that occur in multiple cancer clones involving various organs, including primary and metastatic sites. cfDNA sequencing might thus help to overcome the sampling bias associated with spatial heterogeneity^9–11^. However, it is often difficult to determine the origin of mutant cfDNA. One approach to solving this problem is comparing somatic mutations in cfDNA with those from multiple tumor sites^9,12,13^.

In the present study, we analyzed cfDNA in post-mortem plasma by next-generation sequencing. We further assessed the feasibility of post-mortem cfDNA sequencing and examined the relationship between the mutational status of cfDNA and systemic tumor heterogeneity in a pilot case of prostate cancer. Therefore, we discuss the significance of post-mortem plasma sequencing, the concept of “liquid autopsy,” and the potential of liquid autopsy data to expand the applications of liquid biopsies.

## Results

### Acquisition of cfDNA from post-mortem blood

**Figure 1:**
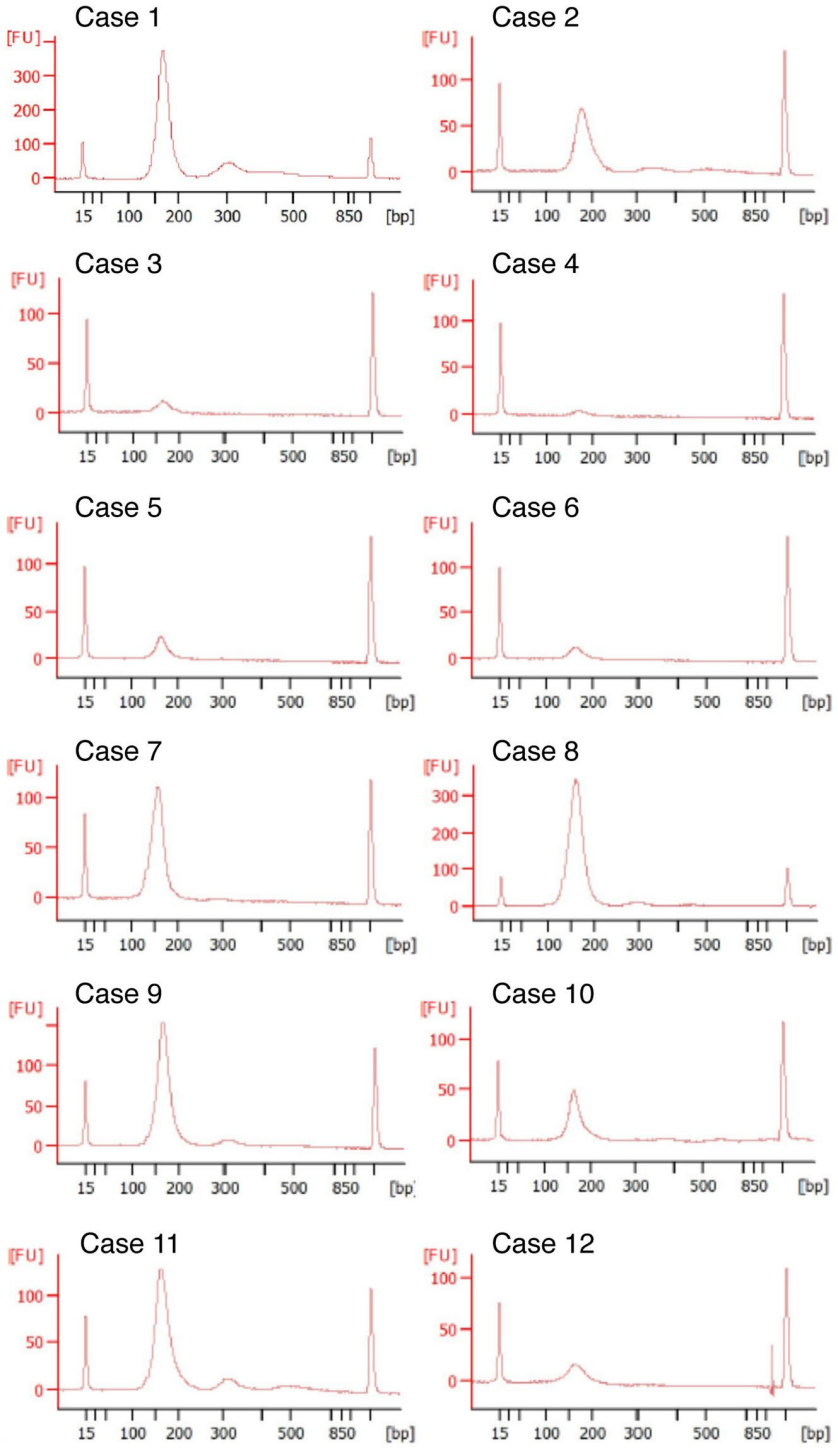
Size distributions of cell-free DNA (cfDNA) extracted from post-mortem plasma. cfDNA extracted from 12 post-mortem plasma samples was analyzed using the Agilent 2100 Bioanalyzer DNA 1000 kit (Agilent Technologies, Santa Clara, CA). The electropherograms showed a fragmented pattern with integral multiples the size of a nucleosome plus linker DNA, which is the typical size distribution of cfDNA.

Real-time PCR-based assays on cfDNA extracted from 12 post-mortem plasma samples revealed the presence of abundant cfDNA (Table 1). The cfDNA samples showed a fragmented pattern with integral multiples the size of a nucleosome plus linker DNA (~167 bp), which is the typical size distribution of cfDNA. Large genomic DNA from normal cells, which makes it difficult to detect somatic mutations with low allele frequencies in cfDNA, was scarcely detected in the cfDNA samples (Fig. 1). In our case series, the presence of residual cancer and time to autopsy did not seem to affect the amount of cfDNA in post-mortem plasma (*P* = 0.6301 and 0.3083, respectively).

**Table 1:** Details of autopsy cases in which post-mortem plasma was sampled.

Case	Age	Sex	Time to autopsy after death	Disease	Viable cancer at autopsy	Plasma cfDNA concentration (ng/mL)
Case 1	58	M	1 hr 21 min	Prostate cancer	Present	2513.4
Case 2	65	M	2 hr 48 min	CNS lymphoma	Present	852.5
Case 3	52	M	6 hr 25 min	MDS	Present	130.0
Case 4	59	F	1 hr 12 min	MAGIC syndrome	Absent	96.3
Case 5	66	M	10 hr 40 min	Glioblastoma	Present	306.9
Case 6	51	F	5 hr 12 min	Hypopharyngeal cancer	Absent	489.5
Case 7	72	M	3 hr 19 min	Gastric lymphoma	Present	1466.4
Case 8	39	M	2 hr 18 min	AML	Present	6549.4
Case 9	35	M	1 hr 42 min	GVHD (AML)	Absent	2815.1
Case 10	43	M	1 hr 19 min	Mycosis fungoides	Present	1055.4
Case 11	38	M	2 hr 53 min	Vertebral artery dissection	Absent	2450.3
Case 12	81	F	11 hr 8 min	Internal carotid artery thromboembolism	Absent	590.0

CNS: central nervous system; MDS: myelodysplastic syndrome; AML: acute myeloblastic leukaemia; GVHD: graft-versus-host disease.

### Clinical history and pathological features of a pilot autopsy case in which extensive genetic analysis was performed (Case 1)

**Figure 2:**
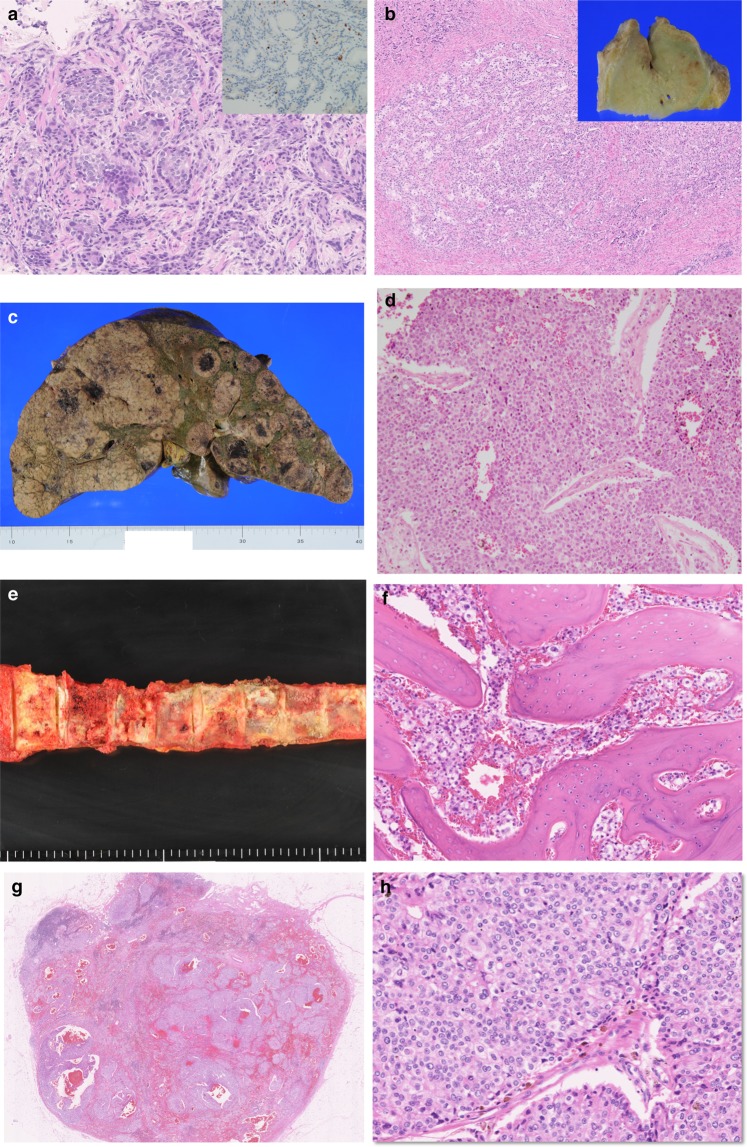
Pathologic features of the prostatic cancer case. (**a**) Prostate core needle biopsy. Histologically, adenocarcinoma with solid and glandular structures was observed. The tumor cells were negative for ERG based on immunohistochemistry analyses (inset). (**b**) Prostate at autopsy. Macroscopically, the prostate was of normal size (inset). Histologically, scattered foci of cancer with degenerative changes were present. (**c**) Liver at autopsy. The liver was extremely enlarged with numerous cancer metastases. (**d**) Histology of the liver metastasis. The metastatic lesion was predominantly composed of poorly differentiated carcinoma growing in solid nests. (**e**) Vertebrae at autopsy. Whitish and sclerotic changes were observed in almost all vertebrae, suggestive of osteogenic metastases. (**f**) Histology of the vertebral metastasis. Thick trabeculae were formed, and the intratrabecular spaces were filled with cancer cells. (**g**,**h**) Histology of the lymph node metastases. Poorly differentiated carcinoma composed of atypical cells with enlarged nuclei growing in sheets was observed.

A 56-year-old male presented with elevated serum prostate-specific antigen (PSA). The prostatic needle biopsy revealed adenocarcinoma (Gleason score, 5 + 4 = 9), which was ERG-negative by immunohistochemistry. Radiographically, multiple bone metastases were detected. The patient received maximum androgen blockade therapy and cytotoxic chemotherapy, which were both ineffective. Later, multiple liver and lymph node metastases developed. The patient died 22 months after the initial presentation due to liver dysfunction. After written informed consent was obtained from family members, an autopsy was performed 1 h and 21 min after death. At autopsy, the liver was enlarged (5605 × *g*), with numerous cancer nodules. Further, osteogenic changes were observed in nearly all vertebrae. Generalised lymphadenopathy was also noted. Histopathological examination revealed small foci of residual adenocarcinoma with degenerative changes in the prostate. Moreover, metastatic foci in the liver, bone, lymph node (cervical, hilar, paraaortic, peripancreatic, etc.), adrenal gland, and lung were identified. All metastases were composed of poorly differentiated carcinoma that grew in solid nests and sheets (Fig. 2).

### Whole-exome sequencing of multiple tumor samples and cfDNA

We selected Case 1 as a pilot case for further genomic analyses because substantial cfDNA was present in the post-mortem plasma and multiple metastases could be evaluated. Somatic mutations that were detected by the WES of eight frozen tumor tissues and the post-mortem plasma cfDNA are shown in Supplementary Table 1. WES of tissues detected 553 somatic mutations. The median number of mutations was 161. We classified these mutations into eight major categories based on commonality (common in 8, 7, 6, 5, 4, 3, and 2 samples and unique to 1). Then, the “unique to 1” category was classified into eight subcategories according to the location of the metastases (Table 2). We designated the “common in 8” category as “trunk mutations”. The mutations in other categories were considered “subclonal mutations,” which accumulated during the clonal evolution of the cancer. In this specific case, approximately 80% of the subclonal mutations belonged to the “unique to 1” category.

**Table 2:** Mutations identified by whole-exon sequencing and their detectability in cfDNA.

Category		No. of somatic mutations		cfDNA detectability%
		Tumor	cfDNA	
Common in 8		71	70	98.6
Common in 7		7	7	100
Common in 6		12	11	91.7
Common in 5		2	0	0
Common in 4		8	7	87.5
Common in 3		17	7	41.2
Common in 2		32	14	43.8
Unique to 1	A	32	5	15.6
	B	40	1	2.5
	N1	25	0	0
	N2	81	16	19.8
	N3	83	0	0
	L1	31	21	67.7
	L2	47	1	2.1
	L3	65	0	0
Total		553	160	28.9

WES of the post-mortem plasma cfDNA was successful and detected 344 somatic mutations, of which 160 were shared with at least one of the metastases. Importantly, nearly all (99%) trunk mutations (“common in 8”) were detectable in the plasma cfDNA. *UBR2* mutation, which was filtered out due to low number of reads in the cfDNA, was the only exception. Detectability of the subclonal mutations was rather low and varied among categories (Table 2).

**Figure 3:**
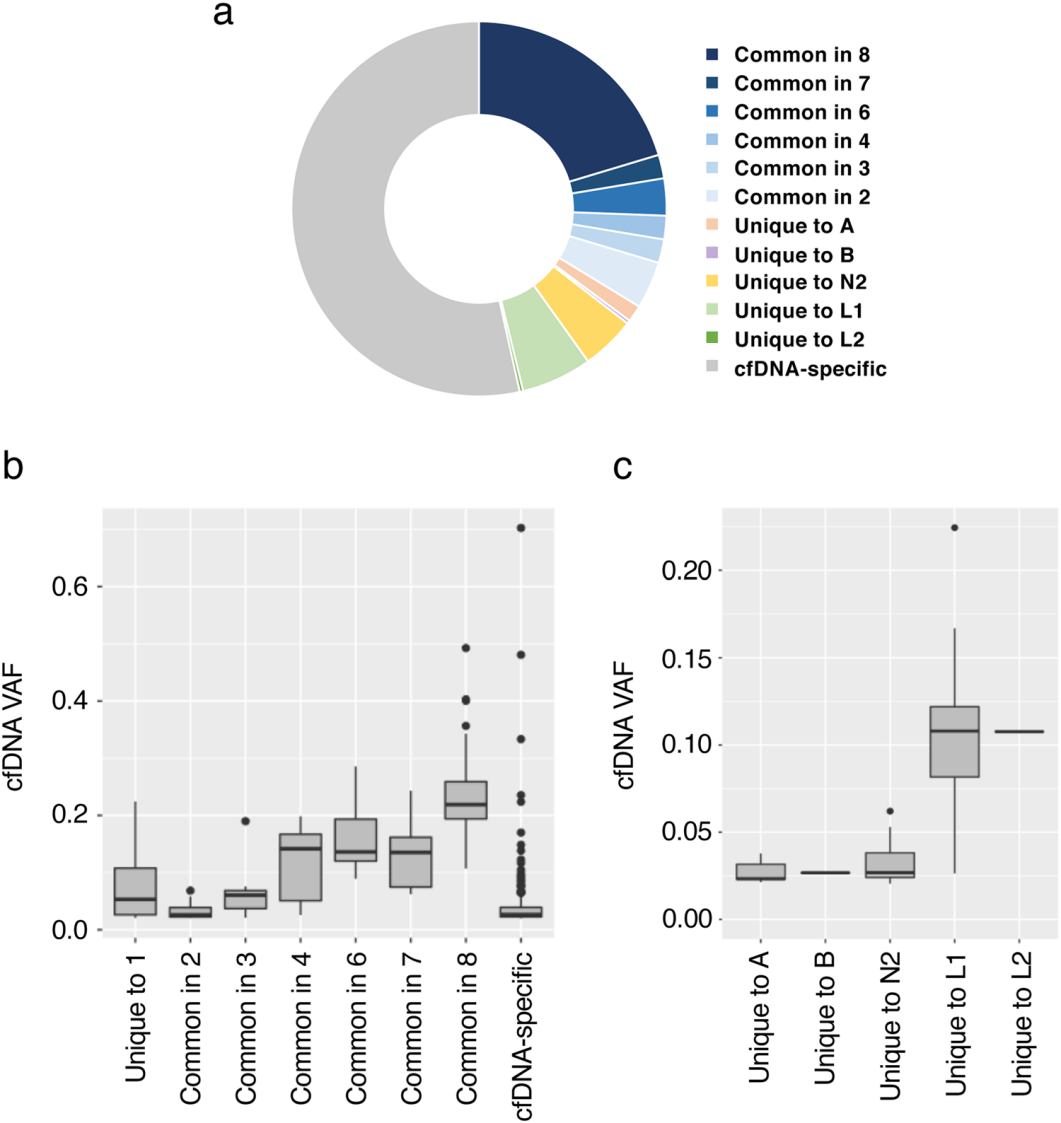
Evaluation of somatic mutations detected in cell-free DNA (cfDNA) by whole-exome sequencing. (**a**) Composition of mutations detected in plasma cfDNA. The classification of mutations was based on whole-exome sequences from eight frozen metastatic tumor tissues (Table 1). This figure is drawn using Excel for Mac (16.16.18) and PowerPoint for Mac (16.16.18), Microsoft Cooperation, Redmond, WA, USA. (**b**,**c**) Variant allele frequencies (VAFs) of mutations detected in cfDNA. Box plots indicate the VAF values of mutations classified by category. Each box shows the median (central line), inter-quartile range (IQR; box), and ±1.5 × IQR (whiskers). Mutations classified as “unique to one” (**b**) were subdivided by tumor sample and are shown in panel (**c**).

The composition of the mutations detected in the plasma cfDNA is shown in Fig. 3a. Mutations identified in at least one of the eight frozen metastases accounted for 47% of the cfDNA mutations, most of which were subclonal mutations. The mutations categorized as unique to A, B, N2, L1, or L2 were also detected in cfDNA, and each comprised 1.5%, 0.3%, 4.7%, 6.1%, and 0.3% of the mutations, respectively. Of note, some cancer-associated gene mutations identified in a single metastatic lesion, such as those in *BRCA2* (c.1318delC, unique to L1), *APC* (c.6053–6071delCAGTTTGTTTCTCAAGAAA, unique to L1), *FBXW7* (c.365C > A, unique to L2), and *PTEN* (c.650T > G, unique to N2), were also detectable in plasma cfDNA. A large fraction (53%) of somatic mutations in cfDNA were cfDNA-specific mutations that were not detected in any of the exome-sequenced metastases (Fig. 3a).

Next, we evaluated VAFs of mutations detected in cfDNA. These tended to correlate with the number of metastatic samples that shared the mutations (Fig. 3b). The VAFs of cfDNA-specific mutations were generally low. However, there were some cfDNA-specific mutations that were detected at relatively high VAF (>0.1) in the plasma. We also investigated the contribution of tumor DNA from different lesions to post-mortem plasma cfDNA by comparing the VAFs of mutations that were unique to a single metastasis. Mutations exclusively detected in a liver metastasis (L1 or L2) had higher VAFs in cfDNA compared to those from metastases of the adrenal gland, bone, or lymph node (*P* < 0.0001; Fig. 3c).

### Sanger sequencing of formalin-fixed paraffin-embedded metastases

**Figure 4:**
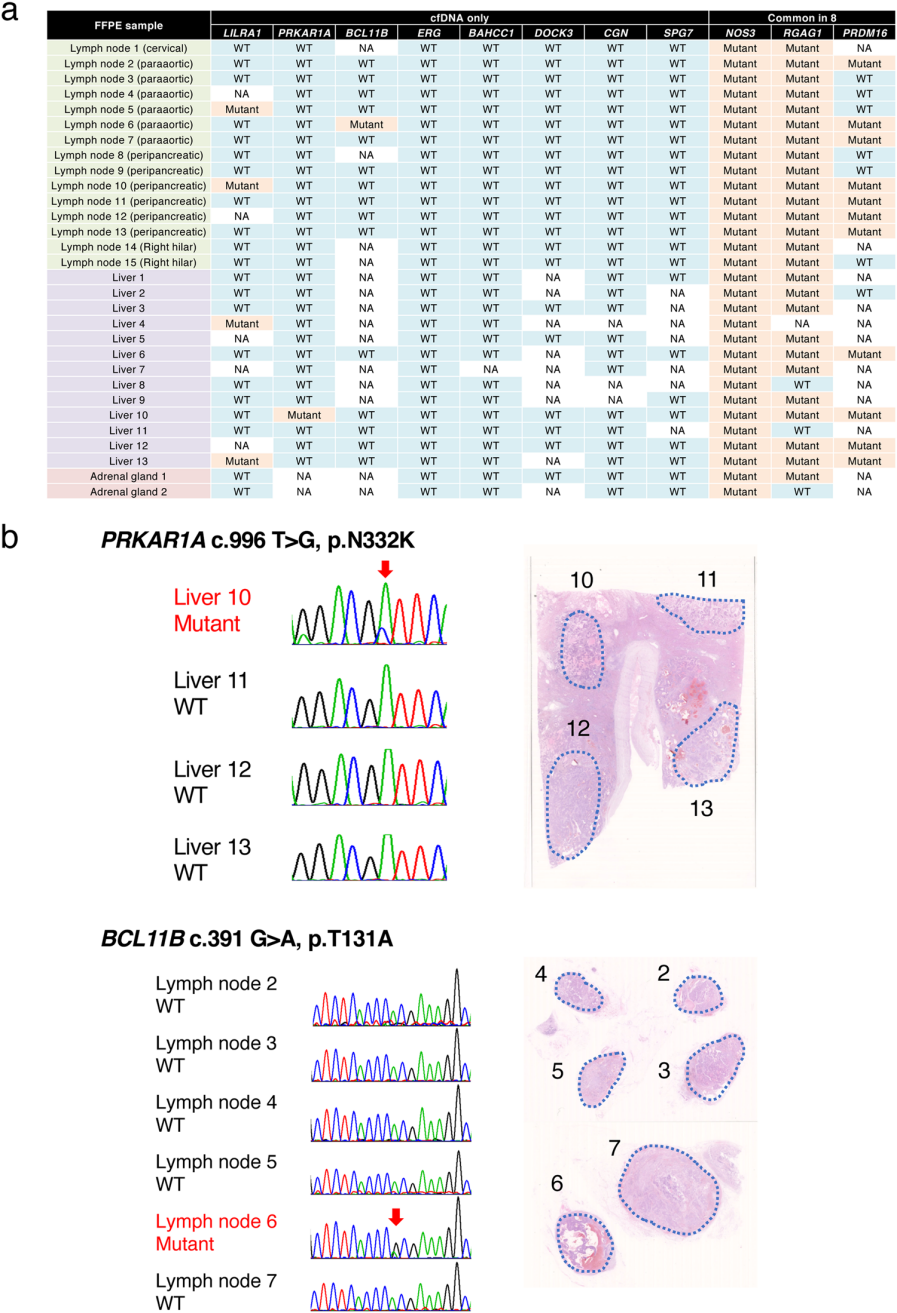
Sanger sequencing of the DNA extracted from formalin-fixed paraffin-embedded metastases. (**a**) Systemic distribution of clones harboring representative cell-free DNA (cfDNA)-specific mutations (N = 8) and mutations commonly detected in eight frozen metastases (N = 3). WT, wild type; NA, data not available due to unsuccessful sequencing. This figure is drawn using Excel 2016 and PowerPoint 2016, Microsoft Cooperation, Redmond, WA, USA. (**b**) One of the cfDNA-specific mutations, *PRKAR1A* (c. 996T > G), was identified in one of the liver metastases (Liver 10). Other metastatic nodules, even those that were in close proximity (Liver 11, 12, and 13), harbored wild-type *PRKAR1A*. Another cfDNA-specific mutation, *BCL11B* (c. 391G > A), was identified in one of the paraaortic lymph node metastases.

In Case 1, more than 50 metastatic lesions were identified in routinely sectioned FFPE specimens. Assuming that clones harboring cfDNA-specific mutations were present somewhere in the body, we performed Sanger sequencing of the representative genes using the DNA extracted from 30 FFPE metastases (Fig. 4a). The cfDNA-specific mutations assessed by Sanger sequencing included *LILRA1* (c.830T > C), which was detected in two lymph node and two liver metastases. Mutations from extremely minor clones, such as those of *PRKAR1A* (c.996T > G) and *BCL11B* (c.391G > A), were also identified in the cfDNA. For example, a *BCL11B* mutation was detected in only one of the lymph node metastases and a *PRKAR1A* mutation was detected in only one of the liver metastases (Fig. 4b). None of the remaining five cfDNA-specific mutations that were analyzed by FFPE-based Sanger sequencing was detected in a metastatic lesion. To determine whether the mutations that we initially designated as trunk mutations were truly “trunk”, we investigated the distribution of clones harboring representative mutations such as *NOS3* (c.1259A > G), *RGAG1* (c.2708G > C), and *PRDM16* (c.346G >T). The *NOS3* mutation was identified in all 30 FFPE metastases that were Sanger sequenced, whereas some of the metastases harbored wild-type *RGAG1* and *PRDM16*.

## Discussion

Concerted efforts for the genomic analyses of cancer have led to the conclusion that cancer is heterogeneous, consisting of multiple clones harboring different sets of genetic changes. The concept of tumor heterogeneity has become increasingly important in the era of precision medicine. Although understanding the molecular variation between tumors is essential for both the development and clinical use of targeted therapies, sampling bias has hindered precise evaluations in this field. This inevitably occurs with biopsy specimens, and even in surgically-resected tissues. Because only a small portion of cancer is sent for sequencing, genotypic assessment to capture systemic tumor heterogeneity is difficult, if not impossible, in living patients. In the present study, we confirmed the potential utility of plasma cfDNA sequencing to overcome the limitations of targeted tissue sampling. The findings are in accordance with those of previous liquid biopsy studies that assessed how systemic cancer heterogeneity is reflected in the plasma cfDNA of the living cancer patients^9,12,13^. Most importantly, in our pilot case, almost all trunk mutations involved in cancer initiation were identified by WES of the plasma cfDNA. In addition, we successfully demonstrated that mutations associated with extremely minor subclones could also be detected in the plasma. Considering that the number of mutations shared by plasma cfDNA and at least one of the metastases (N = 160) was nearly equal to the median number of mutations detected in cancer tissues from eight metastatic sites (N = 161), we can conclude, at least for this specific case, that the sensitivity of plasma cfDNA sequencing is equivalent to or higher than that associated with sequencing a single tumor site when the whole-body mutational landscape of cancer must be examined.

Rapid autopsy is an effective method to investigate the genetic heterogeneity and clonal evolution of cancer. In rapid autopsy, it is generally recommended that the prosector snap-freezes as many cancer samples as possible so that clonal evolution and tumor heterogeneity can be precisely assessed by subsequent genetic analyses such as WES and RNA-seq. However, sampling and sequencing every tumor site is laborious and financially challenging. Consequently, the rapid autopsy program is feasible only at large institutions with sufficient resources.

**Figure 5:**
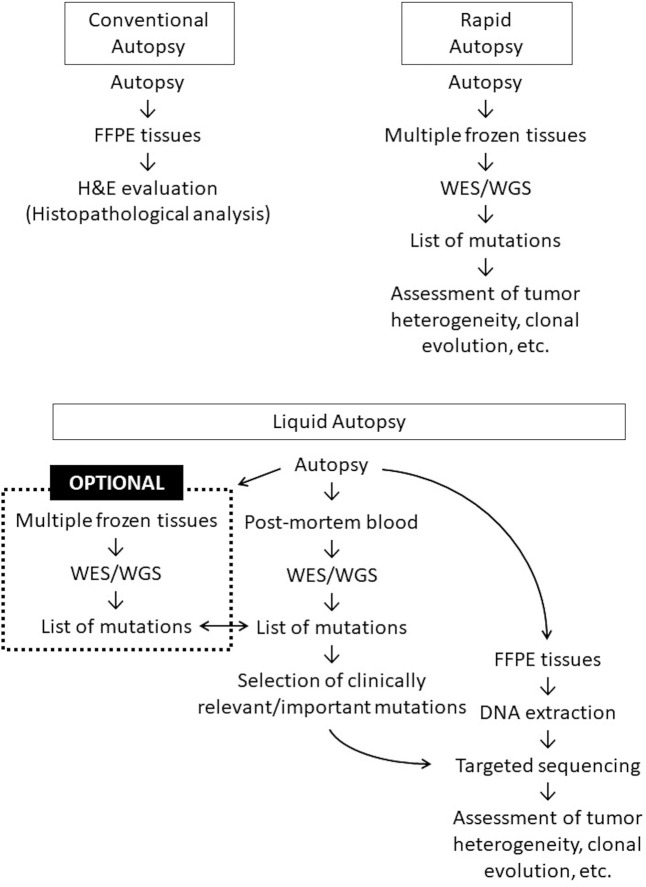
Scheme of liquid autopsy. The concept of liquid autopsy is illustrated and compared to conventional autopsy and rapid autopsy. FFPE, formalin-fixed paraffin-embedded; WES, whole exome sequencing; WGS, whole genome sequencing. This figure is drawn using PowerPoint 2016, Microsoft Cooperation, Redmond, WA, USA.

The present study, based on a pilot case, exposes the limitations of sampling multiple frozen tissues to assess systemic cancer heterogeneity. Our findings suggest that post-mortem plasma cfDNA sequencing, or “liquid autopsy,” is an effective alternative approach (Fig. 5). In combination with FFPE-based targeted sequencing, the clones harboring mutations identified in post-mortem plasma cfDNA can be systemically mapped. Further, post-mortem blood, which is usually disposed of during conventional autopsies, can provide valuable data if sequenced in detail. The preservation of post-mortem blood alone is therefore of value because its genotype can be compared to that of previously obtained biopsies and surgically-resected specimens.

Liquid autopsy also has the potential to elucidate the differential contribution of DNA from different tumor sites to cfDNA. In this case, mutations detected in liver metastases tended to be highly represented in cfDNA compared to those in metastases involving other organs. However, given that no mutations detected in one liver metastasis (L3) were identified in cfDNA, the degree to which tumor DNA contributes to cfDNA could also differ among lesions in the same organ. It should be noted that not all somatic mutations from all known tumor sites were detected in post-mortem cfDNA. The detectability of tumor DNA in cfDNA and the divergence of their VAFs might thus be affected by a variety of factors including tumor size, vascularity, and patterns of invasion. Further investigation is warranted to identify the factors that control the organ-specific contribution of tumor DNA to cfDNA.

This work represents a pilot liquid autopsy study. Post-mortem plasma sampling was performed in a small number of cases, and the genetic analysis data were based on a single case. Technical improvements and standardization of plasma sampling, preservation, sequencing, and other variables are definitely needed. There are limitations to our study, especially regarding interpretation of the results. First, the feasibility of WES using post-mortem plasma cfDNA needs to be validated in a large cohort of cases. Although the amount of cfDNA was successfully quantified in 12 cases, proving its abundance, the quality of the DNA was not assessed. The time limit for plasma sampling after death is a critical issue that requires further investigation. Factors such as post-mortem clot formation surely affect the blood aspiration process. After death, an increase in cellular DNA and cfDNA degradation may occur, resulting in poor sequencing quality. It is also of interest how post-mortem cfDNA differs from cfDNA from living patients. In our study, blood samples were not obtained from the patients before death and, thus, we could not address this issue. Sequential analyses of liquid biopsy samples and a liquid autopsy sample will be informative. It was difficult to identify the factors governing the detectability of tumor mutations in the plasma based on this single case study. Detection patterns of mutations and divergence in their VAFs may vary between the cases, and can be dependent on the primary site of cancer. It is also possible that a specific set of mutated genes are involved in the contribution of DNA from different tumor sites to cfDNA. A gene-specific approach to elucidate the clinicopathological significance of mutated genes in cfDNA is warranted. Although the application of liquid autopsy is discovery-related in nature, we expect that future related studies could provide data that will improve the interpretation of results obtained by liquid biopsy and refine clinical sequencing in living patients.

In summary, post-mortem plasma cfDNA sequencing provides valuable data that cannot be obtained by conventional testing methods, including information on cancer heterogeneity, the systemic distribution of subclones, and the contribution of tumor DNA to cfDNA. We believe that liquid autopsy can be a novel platform for cancer research and a tool to evolve the field of genomic pathology, as it might enable us to narrow down the clones that significantly contribute to patient death. The results of our study suggest that, even in living patients, plasma cfDNA sequencing can yield a large amount of data regarding the mutational status of cancer. The findings broaden the possibilities for liquid biopsy in various clinical contexts.

## Methods

### Post-mortem plasma sampling

We sampled post-mortem plasma from 12 autopsy cases that were enrolled in the Akita Rapid Autopsy Program (ARAP) (Table 1). After obtaining written informed consent from family members, autopsies were performed by the Virchow method. The venous blood was sampled from the inferior vena cava when the heart was removed from the mediastinum. Blood samples were immediately processed to isolate plasma by centrifugation at 3,000 × *g* for 12 min at 4 °C, and the plasma samples were stored at −80 °C until use. The protocol was approved by the institutional review board of Akita University.

### cfDNA extraction from post-mortem plasma

For cfDNA extraction, post-mortem plasma samples were centrifuged at 16,000 × *g* for 10 min at 4 °C to remove cell debris. Cell-free DNA was extracted from 1 mL plasma into 60 µL elution buffer using a QIAamp DNA Circulating Nucleic Acid Kit (QIAGEN) according to the manufacturer’s instructions. Eluted cfDNA was quantified by real-time PCR of human LINE-1 sequences as described in our previous study^14^.

### DNA extraction from tissue samples

During the Case 1 autopsy, tissues from multiple metastases were sampled and snap-frozen. Genomic DNA in eight snap-frozen tissues from metastatic lesions (A, adrenal gland; B, vertebral bone; N1, cervical lymph node; N2, paraaortic lymph node; N3, peripancreatic lymph node; L1, L2, and L3, liver) was extracted using a QIAamp DNA Mini Kit (QIAGEN, Hilden, Germany) according to the manufacturer’s instructions. In addition to tumor tissues, germline DNA was obtained from myocardial tissues to serve as control samples for sequencing analyses. Systemic organs were removed and fixed in formalin and routinely sectioned to prepare paraffin-embedded specimens for histopathological evaluation. For Sanger sequencing, we selected 30 formalin-fixed paraffin-embedded (FFPE) metastases for macrodissection and subsequent DNA extraction. DNA was extracted using a PicoPure DNA Extraction Kit (Life Technologies Corp., Carlsbad, CA, USA).

### Whole-exome sequencing and identification of somatic mutations

Whole-exome sequencing (WES) libraries were prepared using an Agilent SureSelect system and Human All Exon Kit v5.0 (Agilent Technologies, Santa Clara, CA) according to the manufacturer’s instructions. For cfDNA, a sequencing library was prepared using a combination of a KAPA Hyper Prep Kit (KAPA Biosystems, Wilmington, MA) and a SureSelect system as described in our previous study^14^. Massively parallel sequencing was performed on the Illumina HiSeq. 2500 (Illumina, San Diego, CA) platform using a read length of 2 × 100 bp.

Paired-end reads were aligned to the human reference genome (GRCh37) using the Burrows-Wheeler Aligner (BWA) for tumor DNA, cfDNA, and matched germline DNA samples. After probable PCR duplications, for which we removed paired-end reads aligned to the same position, pileup files were generated using SAMtools^15^ and a program developed in-house^16^. To identify somatic point mutations and short indels in cfDNA, stringent confidence filtering conditions were used as previously described^14^, including (i) >8 reads supporting a mutation and (ii) tumor variant allele frequency >0.02. For somatic mutation calling of tumor samples, filtering conditions were as previously described^16^. The mutations identified in the cfDNA were compared to those identified in the metastases. We defined “cfDNA-specific mutations” as mutations not detected in any of the eight metastases sequenced.

### Sanger sequencing of formalin-fixed paraffin-embedded metastases

PCR amplification was carried out using DNA extracted from FFPE metastatic samples, and the primers designed to amplify the mutated regions were randomly selected from the list of cfDNA-specific mutations (Supplementary Table 2). After measuring DNA concentrations, PCR amplification was performed using the T100 Thermal Cycler (Bio-Rad; Hercules, CA). PCR products were sequenced on an Applied Biosystems 3730xl DNA Analyzer (Life Technologies, Carlsbad, CA). The sequencing data were analyzed using Sequence scanner software 2 (Life Technologies, Carlsbad, CA).

### Statistical analysis

The relationship between the time to autopsy and the amount of cfDNA in post-mortem plasma was calculated using the Spearman’s rank correlation test. The significance of differences in variant allele frequencies (VAFs) was tested using Welch’s t-test. All tests were two-sided and P-values < 0.005 were considered statistically significant. All statistical analyses were performed using R (Version 3.3.1, R Development Core Team).

### Ethical issues

All methods were performed in accordance with relevant guidelines and regulations. This study complies with the Declaration of Helsinki and ethical approval was obtained from Akita University, Faculty of Medicine, Ethics Committee. The data were analysed anonymously. Written informed consent from family members was obtained in all the autopsied cases analysed. The permission to use the autopsy samples for this study was obtained from the director of Akita Rapid Autopsy Program.

## Supplementary information

Supplementary Information.

Supplementary Information 2.
