# Supplementary material for: Post-mortem Plasma Cell-Free DNA Sequencing: Proof-of-Concept Study for the “Liquid Autopsy”
Source: Sci Rep. 2020 Feb 7;10:2120. doi: 10.1038/s41598-020-59193-y (PMC7005783; doi:10.1038/s41598-020-59193-y)
Supplement: Supplementary file 2 — Supplementary Information 2. [file 41598_2020_59193_MOESM2_ESM.docx]

**Post-mortem Plasma Cell-Free DNA Sequencing: Proof-of-Concept Study for the “Liquid Autopsy”**

Erina Takai^1^,* Daichi Maeda^2,3^,* Zhuo Li^3,4^, Yukitsugu Kudo-Asabe^3^, Yasushi Totoki^5^, Hiromi Nakamura^5^, Akiko Nakamura^3,6^, Rumi Nakamura^3,6^, Misato Kirikawa^3,6^, Yukinobu Ito^3^, Makoto Yoshida^3^, Takamitsu Inoue^7^, Tomonori Habuchi^7^, Shohei Ikoma^8^, Hiroto Katoh^9,10^, Mamoru Kato^11^, Tatsuhiro Shibata^5,12^, Shumpei Ishikawa^9,10^, Shinichi Yachida^1,5^, Akiteru Goto^3^

1. Department of Cancer Genome Informatics, Graduate School of Medicine, Osaka University, Osaka, Japan.
2. Department of Clinical Genomics, Graduate School of Medicine, Osaka University, Osaka, Japan.
3. Department of Cellular and Organ Pathology, Graduate School of Medicine, Akita University, Akita, Japan.
4. Department of Laboratory Medicine, The First Affiliated Hospital of Xi’an Medical University, Xi’an, Shaanxi, PR China.
5. Division of Cancer Genomics, National Cancer Center Research Institute, Tokyo, Japan.
6. Faculty of Medicine, Akita University, Akita, Japan.
7. Department of Urology, Graduate School of Medicine, Akita University, Akita, Japan.
8. Department of Pathology and Laboratory Medicine, David Geffen School of Medicine, University of California, Los Angeles, CA, USA.
9. Department of Genomic Pathology, Medical Research Institute, Tokyo Medical and Dental University, Tokyo, Japan.
10. Department of Preventive Medicine, Graduate School of Medicine, The University of Tokyo, Tokyo, Japan.
11. Department of Bioinformatics, National Cancer Center Research Institute, Tokyo, Japan.
12. Laboratory of Molecular Medicine, Human Genome Center, The Institute of Medical Science, The University of Tokyo, Tokyo, Japan.

*These authors contributed equally to the work.

**Supplementary Table 2. Primers for Sanger sequencing**

| Primer | Sequence |
| --- | --- |
| LILRA1-F | TGACTTCCTCCAGCTCCCT |
| LILRA1-R | ATCAGGATGTCCAGGGGGTC |
| PRKAR1A-F | CCCATCTTTGCTTTCTCCAG |
| PRKAR1A-R | CGTTTGAGGATGTCTGAGCA |
| BCL11B-F | CTTGGGTGCCTGCTATGAC |
| BCL11B-R | ACCTGCAATGTTCTCCTGCT |
| ERG-F | TTCATTTTGATGTCGCTTTTTG |
| ERG-R | GGCCAGCATTACCTGTGTTT |
| BAHCC1-F | ACAGAGACAACAAAGGGCCG |
| BAHCC1-R | GCTCTGCATCATGAGGTGGG |
| DOCK3-F | CTGTTCCCTCCCTACAGAGC |
| DOCK3-R | GCTGATGGAGCAGTTGTGAA |
| CGN-F | ACCCCACGCATGCTTCT |
| CGN-R | TTCATATGGTCCACACTGCCT |
| SPG7-F | CTGACACAGTTCCCTCCACTC |
| SPG7-R | ACACTGTGTGACGCCGTA |
| NOS3-F | AGGTCTGTGGGTCTGGTTTGAG |
| NOS3-R | TGCTCATTCTCCAGGTGCTTCA |
| RGAG1-F | CATGTGCACACTACCAGTGC |
| RGAG1-R | CACAGTCTCACAGGCTGAGG |
| PRDM16-F | ACATTCCGATCCCAGCAGAC |
| PRDM16-R | TCAATCATACTCAGGCGCGA |
